# Electron-Ion Coupling Mechanism to Construct Stable Output Performance Nanogenerator

**DOI:** 10.34133/2021/9817062

**Published:** 2021-11-09

**Authors:** Yan-Yuan Ba, Jing-Fu Bao, Xin-Tian Liu, Xiao-Wen Li, Hai-Tao Deng, Dan-liang Wen, Xiao-Sheng Zhang

**Affiliations:** School of Electronic Science and Engineering, University of Electronic Science and Technology of China, Chengdu 611731, China

## Abstract

Recently, triboelectric nanogenerators (TENGs) have been promoted as an effective technique for ambient energy harvesting, given their large power density and high energy conversion efficiency. However, traditional TENGs based on the combination of triboelectrification effect and electrostatic induction have proven susceptible to environmental influence, which intensively restricts their application range. Herein, a new coupling mechanism based on electrostatic induction and ion conduction is proposed to construct flexible stable output performance TENGs (SOP-TENGs). The calcium chloride doped-cellulose nanofibril (CaCl_2_-CNF) film made of natural carrots was successfully introduced to realize this coupling, resulting from its intrinsic properties as natural nanofibril hydrogel serving as both triboelectric layer and electrode. The coupling of two conductive mechanisms of SOP-TENG was comprehensively investigated through electrical measurements, including the effects of moisture content, relative humidity, and electrode size. In contrast to the conventional hydrogel ionotronic TENGs that require moisture as the carrier for ion transfer and use a hydrogel layer as the electrode, the use of a CaCl_2_-CNF film (i.e., ion-doped natural hydrogel layer) as a friction layer in the proposed SOP-TENG effectively realizes a superstable electrical output under varying moisture contents and relative humidity due to the compound transfer mechanism of ions and electrons. This new working principle based on the coupling of electrostatic induction and ion conduction opens a wider range of applications for the hydrogel ionotronic TENGs, as the superstable electrical output enables them to be more widely applied in various complex environments to supply energy for low-power electronic devices.

## 1. Introduction

The significance of mechanical energy collection is increasing due to elevated demand for stable and sustainable power sources for the wide-ranging applications of flexible wearable electronics [1] in fields including human-machine interface [2–4], smart skin [5, 6], artificial intelligence [7], wireless sensor networks [8], and the Internet of Things (IoT) [9]. Numerous types of ambient mechanical energy are currently available to us in our living environments in the form of wind [10], water [11], human motion [12], and sound vibration [13]. Based on different mechanisms, including the piezoelectric effect, electromagnetic effect, and electrostatic effect, ambient mechanical energy can be converted to electrical power using the corresponding mechanical energy harvesters, i.e., piezoelectric nanogenerators [14–17], electromagnetic generators [18, 19], and electrostatic nanogenerators [20]. In 2012, a novel power collecting device named as the triboelectric nanogenerator (TENG) was first introduced [21], proving to be a configuration-simple [22], cost-effective [23], and high energy conversion efficiency [24–26] for mechanical energy harvesting [27–29]. According to different types of working mechanisms, TENGs can be categorized into four modes: contact-separation [30–32], single-electrode [33, 34], freestanding [35, 36], and relative sliding [37–39].

Although these four modes of TENGs promote the feasibility of self-powered micro-nano systems in the flexible wearable electronic field, their relatively unstable electrical output performance in dynamic environments limits the practical applications. As a humidity-sensitive phenomenon, triboelectrification is reduced in wet environments, although there is still significant debate regarding the role of a thin moisture layer [40, 41]. Meanwhile, when a TENG is exposed to open air for an extended period of time, its metal electrode becomes oxidized, weakening its ability of transferring charges and thereby reducing the electrical output. It is necessary to eliminate the influence of environmental factors on the stability of TENG output. For example, in order to avoid the adhesion of water molecules to the friction surface, a previous work found polytetrafluoroethylene (PTFE) with hydrophobic properties to effectively function as the friction layer of TENG [42], thereby eliminating the influence of water molecules in the environment on the electrical output of the device. A number of other approaches to reduce environmental interference on TENG have been explored with the aim of realizing the objective of reliable self-powered micro-nano systems [43–45].

As an emerging TENG type, the hydrogel ionotronic TENG [46–48] featuring a hydrogel electrode and dielectric friction layer is based on the combination of the triboelectrification effect and ion conduction, with this TENG attracting growing attention among researchers since it was proposed in 2015 [49]. The special configuration of hydrogel with a kind of three-dimensional (3D) polymeric network allows water molecules to flow freely in the network [50–52], greatly weakening the influence of water molecules on the output of the hydrogel ionotronic TENG. For example, by using gelatin/glycerol and PTFE as triboelectric layers to construct a hydrogel ionotronic TENG, the electrical output of the device can be maintained at higher relative humidity (RH) [53]. However, one primary condition for the normal output of the hydrogel ionotronic TENG is that the hydrogel must contain moisture as the carrier of ion transfer. This constraint makes it difficult to maintain electrical output at a water-free state.

Herein, a flexible stable output performance TENG (SOP-TENG) was proposed based on the coupling of two conductive mechanisms, namely, electrostatic induction and ion conduction. In this work, the SOP-TENG consisted of a calcium chloride-cellulose nanofibril (CaCl_2_-CNF) film and a screen-printed electrode with graphite as the raw material. The CaCl_2_-CNF film, serving as both triboelectric layer and electrode, was fabricated by regenerating carrot tissues that inherited the paramount natural property of plant hydrogel, i.e., ionic conductivity. Additionally, compared with traditional metal electrodes (e.g., copper and aluminum) that are easily oxidized when exposed to air, graphite possesses the excellent chemical stability in the air only to maintain a robustness performance on electrical output of TENG. Furthermore, the effect of electrode size on the electrical output of the SOP-TENG was also comprehensively investigated by comparison with the control group. Thanks to this novel transfer mechanism based on the coupling of electrostatic induction and ion conduction, hydrogel ionotronic TENGs show greater potential for applications in the flexible wearable electronic field since the superstable electrical output enables them to be more widely applied in complex environments to supply energy for low-power wearable electronic devices.

## 2. Methods

### 2.1. Design of the Stable Output Performance Triboelectric Nanogenerator

**Figure 1:**
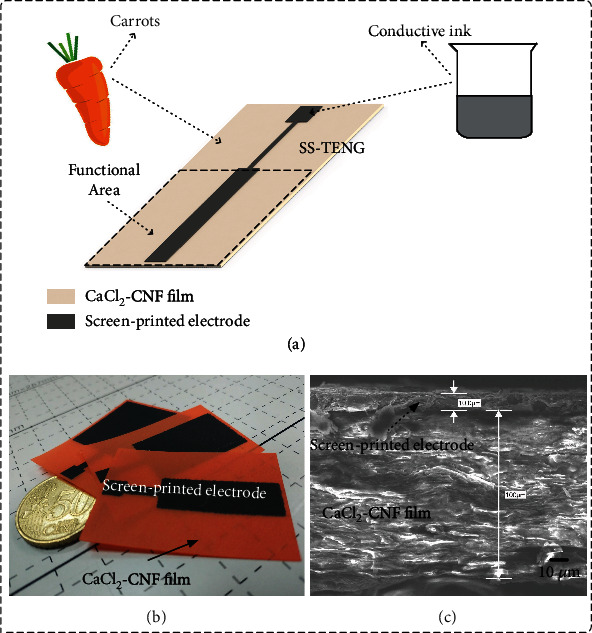
Stable output performance triboelectric nanogenerator (SOP-TENG) based on the coupling of two conductive mechanisms was proposed and fabricated. (a) Schematic illustration of the proposed SOP-TENG reveals its paper-like configuration made of graphite and cellulose nanofibrils extracted from raw carrots. CaCl_2_ was employed to serve as ion sources to realize ion-doped cellulose nanofibril (CNF) films with a thickness of 100 *μ*m, and screen-printed electrodes of different sizes were attached on the surface of CNFs, the photograph of which is shown in (b). (c) Cross-section scanning electron microscopy (SEM) image of the proposed SOP-TENG. More details of preparing cellulose nanofibril film and printing graphite electrode are given in Supplementary Figure S1.

Figure 1(a) shows the illustration of the proposed SOP-TENG which was integrated by the CaCl_2_-CNF film and screen-printed electrode. Using the method presented in our previous work [22], the CaCl_2_-CNF film regenerated from the cellulose tissues of carrots was prepared. The experimental process is briefly summarized as follows. First, the natural cellulose nanofibrils were extracted from the tissues of carrots. For easier extraction, the carrot tissues were boiled in hot water for 8 min to soften the fibers. Then, the processed carrot tissues were mechanically stirred by a blender 20 times to disperse the fibers. It should be noted that the carrot tissues and water were mixed in a weight ratio of 1 : 3 to form original materials for mechanical processing. Subsequently, an 80-mesh filter was introduced to remove large cellulose clusters, making the resulting solution homogeneous. Second, a certain amount of CaCl_2_ with the concentration of 1 mol/L was added into the obtained cellulose solution. Then, the mixture was gradually heated to 200°C and magnetically stirred for 4 hours. Finally, the hybrid gel product was collected in a prepared mold with a glass sheet as the substrate and foam rubber as the baffle to produce the CaCl_2_-CNF film. Here, we used an established technique, namely, roll coating, to flatten the surface of the gel. After curing for 2 hours at 65°C, the final CaCl_2_-CNF film was obtained by peeling off from the mold.

Graphite, an abundant and widely distributed material, has been effectively used due to its excellent electrical conductivity and chemical stability [54]. The introduction of graphite into TENG can solve the problem of electrode oxidation, thereby extending the service life of TENG. Here, the designed screen-printed electrode made of graphite was printed onto one surface of the CaCl_2_-CNF film using screen-printing technology. During this process, the number of the steel mesh apertures is 200, and the graphite ink was purchased from Jujo Chemical Co., Ltd. A dispersant purchased from Zhongyi Ink & Paint Co., Ltd. was also needed, allowing the graphite to pass through mesh easily. The final hybrid ink containing the graphite ink and dispersant in a weight ratio of 10 : 1 was dried at 65°C in an oven for 20 min to form the electrode of SOP-TENG. The block resistance of the screen-printed electrode was measured to be 28.1 *Ω*/sq using a multifunction digital four-probe tester. The process flowchart of the proposed SOP-TENG is shown in Figure S1. It is worth mentioning that all electrical performance tests in this article were limited to an area of 3 × 3 cm^2^, as the black dashed box shown in Figure 1(a). Figure 1(b) shows three SOP-TENGs with different sizes of electrodes, namely, the 1/3 size, 2/3 size, and full-size electrode SOP-TENGs. Figure S2 shows the flexibility of the CaCl_2_-CNF film [17]. In the previous work, a cellulose nanofiber-based TENG consisting of two CNF films as the dielectric layer and the silver nanowire (AgNW) as electrodes which showed potential to be an e-paper-like device was proposed [41]. Compared with this previous CNF-based TENG, the SOP-TENG possesses advantages of simpler process and greater environmental adaptability. Meanwhile, the thickness of CaCl_2_-CNF film and the screen-printed electrode is 100 *μ*m and 10 *μ*m, respectively, as shown in Figure 1(c). The ultrathin configuration enables SOP-TENG to show a promising application potential in the wearable electronic field as it can be easily integrated with other electronic components.

**Figure 2:**
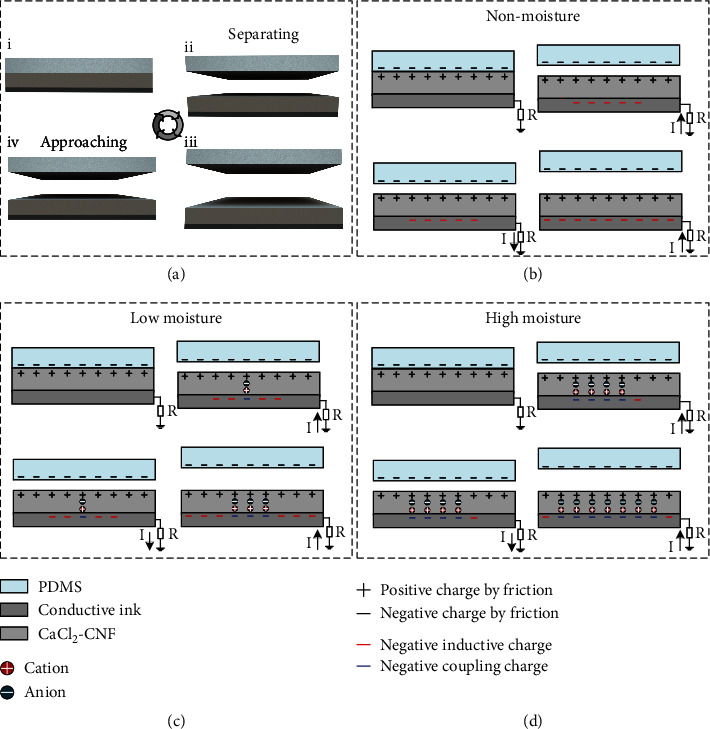
Schematic view of the coupling of two conductive mechanisms of the SOP-TENGs with different moisture contents. (a) Demonstrated the four important processes of the designed SOP-TENG in one contact-separation cycle, namely, (i) the state of triboelectric pairs (i.e., PDMS and CaCl_2_-CNF film) in contact with each other, (ii) the process of gradual separation of the triboelectric pairs, (iii) the state of the triboelectric pair separation to the maximum, and (iv) the process of triboelectric pairs gradually approaching each other until they touch each other. Schematic diagrams of charge transfer in the four working processes of the SOP-TENG were shown when the CaCl_2_-CNF film contained (b) nonmoisture, (c) low moisture, and (d) high moisture.

For the general hydrogel ionotronic TENG, a hydrogel layer is packaged in an elastomeric cell and connected to the load circuit by a bonding wire [47, 48]. When a contact-separation process occurs between a dielectric and the elastomer cyclically, ions transfer in the hydrogel due to the effect of electrostatic charges generated by the triboelectrification effect, and the capacitive coupling of the electric double layer (EDL) causes the electrons to transfer between the bonding wire and the ground, generating an alternating current [51]. The EDL acts as a capacitor with high capacitance and couples the ionic current in the hydrogel and the electric current in the electrode. The working mechanism of the hydrogel ionotronic TENG is schematically illustrated in Figure S3. In contrast to the traditional hydrogel ionotronic TENGs requiring moisture and a hydrogel layer as the carrier for ion transfer and the electrode, respectively, the proposed SOP-TENG using a CaCl_2_-CNF film as a friction layer effectively realizes a superstable electrical output under varying moisture contents (i.e., nonmoisture, low moisture, and high moisture) because of the coupling of electrostatic induction and ion conduction. The SOP-TENG is best suited to work in single-electrode mode for its simple structure, and the four important processes (i.e., (i) initial status, (ii) separating status, (iii) maximum separation status, and (iv) approaching status) of the device in one contact-separation cycle are schematically illustrated in Figure 2(a). While the surface with the non-screen-printed electrode of CaCl_2_-CNF film acted as friction surface, a PDMS film with a size of 3 × 3 cm^2^ attached on the shaker served as the corresponding triboelectric layer. In step (i), an external force generated by the shaker made the PDMS film fully in contact with the CaCl_2_-CNF film, leading to the contact electrification between two surfaces. Due to different material properties, CaCl_2_-CNF and PDMS surfaces accumulated positive and negative charges, respectively. In step (ii), the PDMS film gradually moved away from the CaCl_2_-CNF film. In order to maintain the overall electrostatic balance of the SOP-TENG, the negative charges in the ground flows to the screen-printed electrode through the external load circuit. When the distance between CaCl_2_-CNF and PDMS films could not be increased anymore, the separation process ended and the surface charge density of SOP-TENG reached its maximum value simultaneously (step (iii)). If the PDMS film approached the CaCl_2_-CNF film again, electrons resume their flow but in the reverse direction (step (iv)).

When the CaCl_2_-CNF film was in the nonmoisture status, the ions, namely, Ca^2+^ and Cl^−^, could not diffuse due to the lack of the carrier, thereby the charge transfer only relied on the effect of electrostatic induction. However, the moisture appearing in the CaCl_2_-CNF film realized the charge transfer by the coupling of electrostatic induction and ion conduction. Meanwhile, with the enhancement of moisture content in the CaCl_2_-CNF film, the role of ion conduction was gradually increasing in these two conductive mechanisms. The electron transfer mechanisms in the four important processes are shown in Figures 2(b)–2(d). Figures 2(b)–2(d) show the electron transfer when the CaCl_2_-CNF film was in the nonmoisture, low moisture, and high moisture status, respectively. It must be pointed out that the surface charge density was only related to the material, so there was no difference on the charge density in three moisture contents of the CaCl_2_-CNF film. In Figure 2(b), the CaCl_2_-CNF film had no ionic conductivity due to the lack of the moisture participation; the charges accumulated on the screen-printed electrodes were induced by the electrostatic induction. In other words, the SOP-TENG in this moisture content status can be considered as a traditional single-electrode TENG. In Figure 2(c), the low moisture content made the CaCl_2_-CNF film has a slight ionic conductivity. However, the dielectric constant of CaCl_2_-CNF film increased due to the immersion of moisture which reduced the effect of electrostatic induction. This part of the induced charges in the screen-printed electrode will be replaced with the coupling charges generated by the ion conduction. The increasing of moisture content improved the ionic conductivity of the CaCl_2_-CNF film which further weakened the electrostatic induction effect, resulting in an increase in the proportion of the coupling charges in the screen-printed electrode, as shown in Figure 2(d).

### 2.2. Tests and Measurements

To investigate the electrical performance of the SOP-TENG in a comprehensive method, a waveform generator (33250A, Agilent), a power amplifier (YE5872A, Sinocera Piezotronics, Inc.), and a modal shaker (JZK-10, Sinocera Piezotronics, Inc.) were assembled to constitute a magnitude and frequency controllable force output system (i.e., vibration platform). The output current and voltage of SOP-TENG were tested by a low-noise current preamplifier (SR570, Stanford Research Systems) and a digital oscilloscope (DS2302A, RIOGL), respectively. The amount of charge transferred in one cycle was measured by an electrometer (6514, Keithley). The cross-section image of the SOP-TENG was investigated by an electron-scanning microscope (JSM-7600F, JEOL). To cure the CaCl_2_-CNF film and the screen-printed electrode, a vacuum drying oven (PVD-050C, Shanghai Shibei Instrument Equipment Co., Ltd., China) was introduced. A blender (769S, HATTIECS) was used to disperse the fibers of the carrot tissues. A magnetic mixer machine (B11-2, Shanghai Sile Instrument Co., Ltd., China) was used to stir and heat the hybrid cellulose solution.

## 3. Results and Discussion

### 3.1. Output Performance of the SOP-TENG

**Figure 3:**
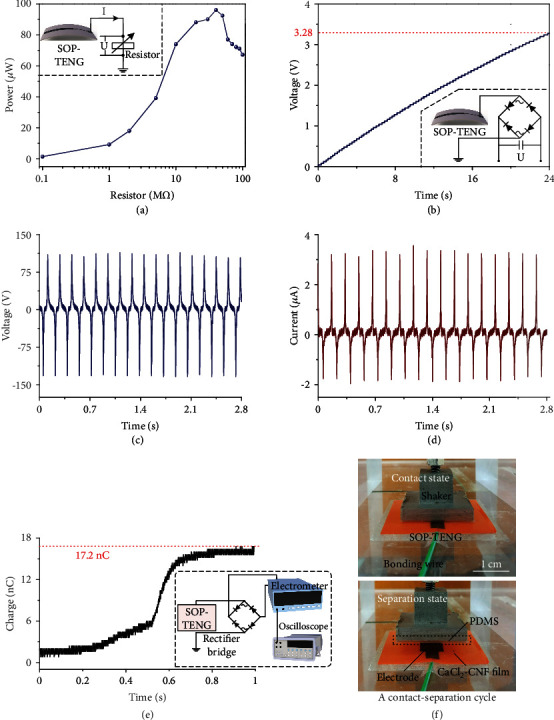
Electrical measurements of 3 × 2 cm^2^ screen-printed electrode size SOP-TENG using a 6 Hz stable vibration platform. (a) Power output on the resistor (i.e., load) connecting to the fabricated SOP-TENG. When the resistance was raised from 0 to 100 M*Ω*, the power value increased at first and then dropped. The power was maximized for a 40 M*Ω* external resistance, which equals the internal resistance of SOP-TENG. (b) The voltage profile of a 1 *μ*F capacitor was measured when charging by SOP-TENG, revealing the mean power of the device. (c, d) Voltage and the current waveforms of the SOP-TENG, respectively. (e) Electrometer and oscilloscope measured the amount of charge transferred by the fabricated SOP-TENG via a full-wave rectifier bridge during (f) one contact-separation cycle.

As a single-electrode mode TENG, the fabricated SOP-TENG needs a dielectric layer to form triboelectric pairs. A proposed electron-cloud-potential-well model based on the electron-emission-dominated charge-transfer mechanism in previously published studies can be applied to explain the physical mechanism of the triboelectrification in conventional materials. This model reveals that the deeper potential well makes the cellulose to be a triboelectric material with positive polarity. To obtain high output performance of the SOP-TENG, poly(dimethylsiloxane) (PDMS) is selected as another triboelectric layer for its negative polarity due to the lower potential well. The electrical performance of the proposed SOP-TENG is systematically evaluated by a fixed force with the frequency of 6 Hz (Figure 3), which is supported by a vibration platform consisting of a modal shaker, a signal generator, and a power amplifier.

To explore the impact of resistance on the power output of the SOP-TENG, resistors with values ranging from 0.1 M*Ω* to 100 M*Ω* were connected to the TENG in series. Figure 3(a) illustrates the relationship between the power output characteristic and external load resistance. With 40 M*Ω* load resistance, the instantaneous power reached the maximum power output value of 96 *μ*W. Figure 3(b) shows that a commercial capacitor with 1 *μ*F was charged by the SOP-TENG and the inset was the experimental setup with a bridge rectifier. It could be seen that within 24 s, the voltage of a 1 *μ*F capacitor could reach up to 3.28 V at a frequency of 6 Hz, which exhibited the superior electrical output capacity of SOP-TENG. The peak-to-peak voltage and short-circuit current of the SOP-TENG reached 246 V and 5.52 *μ*A, respectively, as shown in Figures 3(c) and 3(d). To further elevate the performance of the SOP-TENG, its generated charge was measured by an electrometer, as shown in Figure 3(e). From Figure 3(f), in one contact-separation cycle, the amount of charge generated by the SOP-TENG was 17.2 nC.

### 3.2. Effect of the CaCl_2_-CNF Film on Stable Output Performance

The cellulose nanofibril (CNF) is the most abundant natural polymer on earth, which attracts much attention in the field of TENG for its advantages of being widely accessible, cost-competitive, and biodegradable. In the previous research work, both treated and untreated CNFs were used as raw materials for fabricating TENGs. However, the CNF-based TENGs were difficult to maintain a stable output in a dynamic environment due to the complex composition of CNF, which limited the further research on its properties, such as ion channels and bioelectricity. In this article, we avoided the above-mentioned interference through the coupling of electrostatic induction and ion conduction, so that the TENG with a superstable output was obtained.

**Figure 4:**
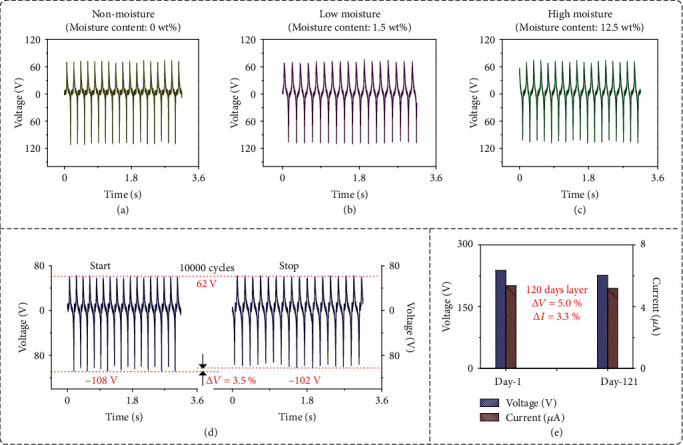
Study of effects of CaCl_2_-CNF film parameters on the output stability of SOP-TENG. (a–c) Study of the effect of moisture content on the output voltage of the SOP-TENG. (a) Voltage waveform of SOP-TENG with nonmoisture content. (b) Voltage waveform of SOP-TENG with low moisture content. (c) Voltage waveform of SOP-TENG with high moisture content. (d) Study of the effect of continuous operation on the output voltage of the SOP-TENG. The voltage data gained through the oscilloscope only dropped by 3.5% after continuous operation of 10 000 cycles, conforming superstable output performance of the SOP-TENG. (e) Study of the effect of device aging on the voltage output performance of the SOP-TENG. The measured voltage and the current response of a SOP-TENG after 120 days were 226 V and 5.18 *μ*A, respectively, which only declined by about 5% and 3.3% compared with the initial value.

Figure 4 shows the influences of the changes in CaCl_2_-CNF film parameters on the stable output of the proposed SOP-TENG, which included moisture content, continuous operation, and device aging. Figures 4(a)–4(c) demonstrate voltage output waves of the SOP-TENG under nonmoisture, low moisture, and high moisture statuses, respectively. Here, we named 0 wt% moisture content as the nonmoisture status, 1.5 wt% moisture content as the low moisture status, and 12.5 wt% moisture content as the high moisture status. The peak-to-peak voltages of the SOP-TENG with nonmoisture (Figure 4(a)), low moisture (Figure 4(b)), and high moisture (Figure 4(c)) statuses were 186 V, 186 V, and 182 V, respectively. These values proved the robustness of transferring electrostatic charges of SOP-TENG by coupling of the two transmission mechanisms. In addition, the voltage output curves of SOP-TENGs at 30 RH%, 50 RH%, 70 RH%, and 90 RH% were added to further enhance the reliability of the research, as shown in Figure S4.

Figure 4(d) shows the influence of continuous operation on the voltage output performance of the SOP-TENG. It is worth noting that the voltage output of SOP-TENG just dropped by 3.5% after running 10 000 times. This phenomenon showed that the CaCl_2_-CNF film possessed the good mechanical strength to avoid the damage by friction, because of which the superstable output of the SOP-TENG was maintained.

Figure 4(e) shows the influence of the device-aging on the voltage output performance of the SOP-TENG. For this test, a SOP-TENG was placed in an indoor environment for 120 days with about 70% relative humidity at room temperature. There was no visible aging on the friction surface of the SOP-TENG. Meanwhile, the screen-printed electrodes using graphite as raw materials still had the excellent electrical conductivity due to their chemical stability. The measured voltage and the current response of the SOP-TENG after 120 days were 226 V and 5.18 *μ*A, respectively, which only declined by about 5% and 3.3% compared with the initial value of 238 V and 5.36 *μ*A. It can be concluded that through the above long-term test, the output performance of the fabricated SOP-TENG kept highly consistent. The hydrogel absorbs moisture and swells. Here, an experiment was designed to explore whether the electrical properties of a swollen device would be reduced after drying. First, the SOP-TENG was humidified by a humidifier and then dried in an oven at 80°C for 2 hours. The output voltages were normalized according to the initial voltage value. The normalized voltage value after 2 hours of 80°C drying is shown in Figure S5.

### 3.3. Effect of the Relative Humidity on Stable Output Performance

Natural CNF shows excellent hydrophilia and hydroscopicity due to its physical and chemical properties inheriting from the plant hydrogel. The large amount of -OHs contained in the macromolecules of natural CNF is capable of generating the coordination bonds with H_2_O molecules, which makes CNF hydrophilic. Meanwhile, the nanolevel gaps widely existing in the CNF film produce a strong capillary action which endow the CNF film with excellent hydroscopicity. Also, the CNF's amorphous area and CaCl_2_ molecule can absorb and store the attaching moisture on the surface of CNF film due to the physical property of the amorphous area's loose tissue and chemical property of CaCl_2_'s hydration, respectively. With hydrophilia and hydroscopicity, SOP-TENG is expected to avoid the influence of the water molecules from the environment.

To examine the humidity effect on SOP-TENG, a comprehensive investigation system with a humidity detection platform, a vibration platform, and an electrical measurement platform was established, as shown in Figure S6 and Supplementary Video S1. A sealed box with a volume of 50 cm × 40 cm × 40 cm made of polymethyl methacrylate (PMMA) was introduced to limit the change of the environmental relative humidity in a small space, so that the measurement of the output of the SOP-TENG would be more convenient and accurate. In the meantime, a hygrometer was used to reflect the variational value of RH affected by the vapor generated by a humidifier. The acrylic box, humidifier, and hygrometer formed the humidity detection platform. The vibration platform was established to supply a controllably compressive force with the designed frequency, which consisted of a waveform generator, an amplifier, and a shaker. A digital oscilloscope and a low-noise current preamplifier formed the electrical measurement platform to test the voltage and current output performance of the SOP-TENG, respectively. Based on this comprehensive investigation system, the data on the electrical output affected by the relative humidity were given as follows.

**Figure 5:**
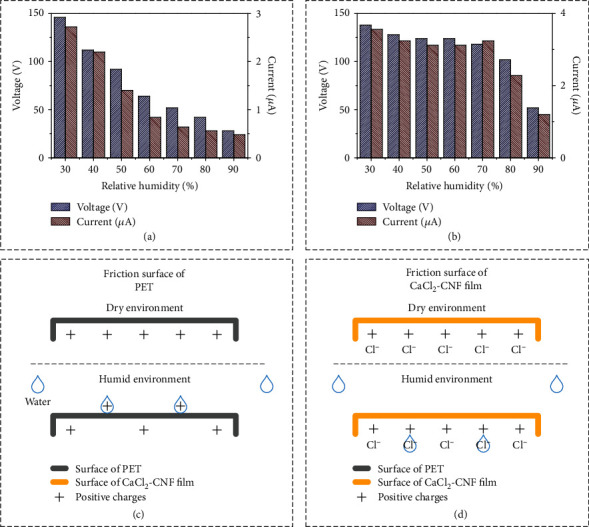
Study of the effect of the relative humidity on output stability of SOP-TENG/PET-based TENG. (a) The influence of relative humidity on the electrical performance of the PET-based TENG. As the relative humidity increased, the voltage and current output of the PET-based TENG decreased rapidly. (b) The effect of relative humidity on the electrical performance of the SOP-TENG. Before the relative humidity rose to 80%, it had a slight effect on the electrical output performance of SOP-TENG. (c, d) Showed the electrostatic charge distribution on the friction surfaces of PET and CaCl_2_-CNF film in the dry and humid environment, respectively.

In the experiment, a single-electrode TENG with PET as the friction layer was used as a control group.  Figure 5(a) describes the variation results of the PET-based TENG's electrical output with the RH in the PMMA box when the screen-printed electrode size was 3 × 3 cm^2^, which showed that the electrical output decreased rapidly as the elevation of RH. The PET-based TENG achieved the maximum voltage and current outputs of 146 V and 2.72 *μ*A at 30% RH. However, when the RH in the PMMA box rose to 70%, the output voltage of the PET-based TENG dropped to 52 V, losing 64% of the output performance. Compared with the PET-based TENG, the SOP-TENG presented better robustness performance on the electrical output with CaCl_2_-CNF film serving as a triboelectric layer, as shown in Figure 5(b). Given the maximum voltage of 138 V generated at 30% RH, it is clearly indicated that using the CaCl_2_-CNF film as the friction layer realizes a superstable output voltage of 118 V when RH changed to 70% due to the excellent hydrophilia and hydroscopicity of the film.

The reasons behind the different electrical output performances between the PET-based TENG and SOP-TENG were worth discussing. With the enhancement of RH, the density of water molecules in the air increased, resulting in an increasing density of water molecules on the surface of the TENG's friction layer. For PET-based TENG, the water molecules would remain on the friction surface and absorb electrostatic charges, thereby decaying the electrostatic charge density which was positively related to the electrical output, as shown in Figure 5(c).  Figure 5(d) shows the electrostatic charge distribution on the friction surface of the SOP-TENG under different environmental conditions. The excellent hydrophilia and hydroscopicity enabled the CaCl_2_-CNF film to absorb the water molecules on the surface, resulting in a constant charge density to produce the robust output of SOP-TENG.

### 3.4. Effect of the Electrode Size on Stable Output Performance

The screen-printed electrode, as an essential component of the SOP-TENG, transfers the charges generated by the coupling of electrostatic induction and ion conduction to a capacitor that is used to power the low-power electronic devices. The screen-printed electrode is sensitive to water molecules, as the above phenomenon occurs on the surface of the PET-based TENG. An effective strategy to solve this problem is to encapsulate the screen-printed electrode. However, the SOP-TENG sacrifices advantages of simple configuration, leaner process flow, and competitive cost when using this method, so that the massive production of the SOP-TENG was restricted. Based on the principle of the edge effect of the capacitor, reducing the electrode size of the single-electrode TENG by 2/3 has little effect on its electrical output. The output of SOP-TENGs with different electrodes in the nonmoisture status was in complete agreement with the above theory, as shown in Figure S7. Therefore, reducing the electrode size of the SOP-TENG is an effective approach to maintaining the output performance in the humid environment. However, the reduction degree of the electrode size is limited. When the electrode size is much smaller than the friction surface, the electrical output performance of the device will be greatly reduced because the electrode can not interact the static charge at the distance friction area. The data in Figure S7 demonstrate this phenomenon. When the electrode area is smaller than 3 × 0.5 cm^2^, both the voltage and the current of SOP-TENG begin to drop sharply.

**Figure 6:**
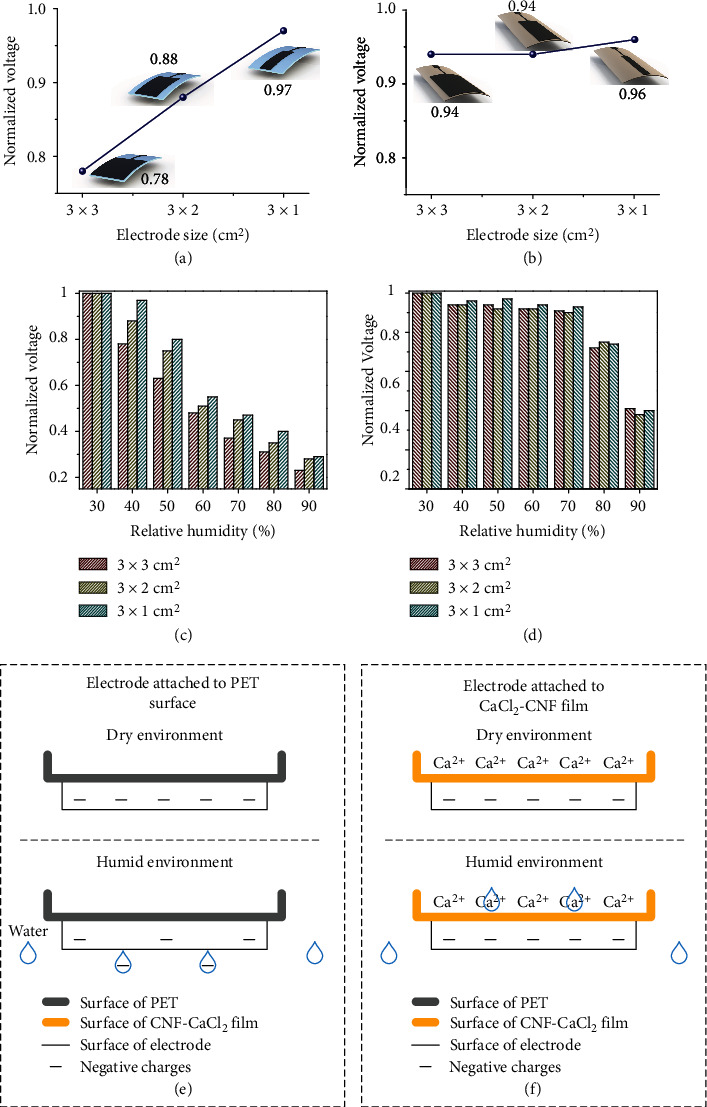
Study of the effect of the electrode size on the output stability of the SOP-TENG. (a) Normalized voltage generated from the PET-based TENGs with different electrode sizes (i.e., 3 × 3 cm^2^, 3 × 2 cm^2^, and 3 × 1 cm^2^) at 40% RH. The output voltages were normalized according to those of the respective PET-based TENG at 30% RH. (b) Showed the effect of electrode size on the normalized voltage of the SOP-TENG. The electric outputs were measured at 40% RH. The output voltages were normalized according to the voltage outputs of the respective SOP-TENG at 30% RH. (c, d) Systematic comparison of the normalized voltage generated from the PET-based TENGs and SOP-TENGs with different electrode sizes at different RH levels, respectively. (e, f) Showed the induced charge distribution on the surfaces of electrodes in the dry and humid environment, respectively.

Figure 6(a) shows the effect of the screen-printed electrode size on the normalized voltage of the PET-based TENG. It must be noted that the output voltages were normalized according to those of the respective TENG at 30% RH, since the main purpose of our experiment was to examine the output stability of the device at different environment RH. The normalized voltages of the PET-based TENGs with electrode sizes of 3 × 3 cm^2^, 3 × 2 cm^2^, and 3 × 1 cm^2^ were 0.78, 0.88, and 0.97, respectively, at 40% RH. Moreover, the reduction of two-thirds electrode size increased the average normalized voltage of the PET-based TENG by 10%, as shown in Figure S8a. The result showed that there was a negative correlation between the electrode size of the PET-based TENG and relative humidity. For SOP-TENGs, the reduction of the electrode size also led to good results, in spite of the improvement of normalized voltage being negligible. The normalized voltages of 0.94, 0.94, and 0.96 corresponded the electrode sizes of 3 × 3 cm^2^, 3 × 2 cm^2^, and 3 × 1 cm^2^ of the SOP-TENGs, respectively, as shown in Figure 6(b). Also, the reduction of two-thirds electrode size increased the average normalized voltage of the SOP-TENG by 2%, as shown in Figure S8b. Figures 6(c) and 6(d) show the normalized voltages of the PET-based TENGs and the SOP-TENGs with three electrode sizes under different levels of RH conditions (i.e., 30%, 40%, 50%, 60%, 70%, 80%, and 90%). In the meantime, the voltage of these two kinds of TENGs with three electrode sizes with changing RH is shown in Figure S9.

Further analyses of the effect of electrode size on electrical output are shown in the following. The density of water molecules in the PMMA box increased with the enhancement of RH, resulting in an increasing density of the water molecules on the surface of the screen-printed electrode. For the PET-based TENG, the water molecules had the tendency to remain on the surface of the screen-printed electrode to absorb the charges generated by the coupling of electrostatic induction and ion conduction and thereby decay the electrical output, as shown in Figure 6(e). According to this phenomenon, shrinking the electrode area of the PET-based TENG can reduce the number of charges absorbed by water molecules, thereby achieving the purpose of increasing the normalized voltage, as shown in Figure S10. Figure 6(f) shows the charge distribution on the screen-printed electrode of the SOP-TENG under different environmental conditions. The CaCl_2_-CNF film exhibited its hydrogel properties in the humid environment, thereby the coupling of electrostatic induction and ion conduction will counteract the effects of the relative humidity. This theory showed that the influence of electrode size on the SOP-TENG was extremely weak, revealing a superstable output performance of the device.

## 4. Conclusion

In this work, a flexible stable output performance TENG (SOP-TENG) with a CaCl_2_-CNF film and a screen-printed electrode was proposed. This kind of SOP-TENG combines electrostatic induction and ion conduction to realize a new conductive mechanism. The cellulose nanofibril (CNF) film fabricated by regenerating the carrot tissues inherits the ionic conductivity of the plant hydrogel and acts as both triboelectric layer and electrode. Moreover, the CaCl_2_ was doped into the CNF film to constitute the CaCl_2_-CNF film with a superior conduction property. Meanwhile, graphite was introduced to form the screen-printed electrode for its stable chemical properties. The proposed SOP-TENG demonstrated the outstanding output performance with the peak-to-peak voltage and short-circuit current of 246 V and 5.52 *μ*A, respectively. The robustness of the SOP-TENG was mainly researched from three aspects, namely, effect of the CaCl_2_-CNF film, effect of the relative humidity, and effect of the electrode size. In terms of the effect of the CaCl_2_-CNF film, the influences of moisture content, durability, and device aging on the output of the SOP-TENG were fully investigated. A comprehensive investigation system with a humidity detection platform, a vibration platform, and an electrical measurement platform was established to test the effect of the relative humidity on the stable output of the SOP-TENG. And the effect of the electrode size on the output of the SOP-TENG was also investigated comprehensively with the electrical measurements and theoretical analysis. The experimental data also corroborated that the SOP-TENG had a superstable electrical output in various parameters due to the compound transfers of ion and electron. In summary, with this novel working mechanism based on the coupling of electrostatic induction and ion conduction, SOP-TENG shows greater potential to supply energy for flexible wearable electronics in a complex environment.

## Data Availability

The data used to support the findings of this study are available from the corresponding author upon reasonable request.

## Supplementary Materials

Supplementary 1 Figure S1: fabrication process flowchart of the stable output performance triboelectric nanogenerator (SOP-TENG) based on the composite of the CaCl_2_-CNF film and the screen-printed electrode. Figure S2: a photograph showing the flexibility of the calcium chloride-cellulose nanofibril (CaCl_2_-CNF) film. Figure S3: schematic view of the work mechanism of the general hydrogel ionotronic TENG. Figure S4: the voltage output curves of SOP-TENGs at (a) 30 RH%, (b) 50 RH%, (c) 70 RH%, and (d) 90 RH%. Figure S5: the initial normalized voltage, normalized voltage after the swelling, and normalized voltage during the swelling of the SOP-TENG. Figure S6: a comprehensive investigation system for studying the influence of humidity on the electrical output of the SOP-TENG was established, which included a vibration platform, a humidity detection platform, and an electrical measurement platform. Figure S7: electrical output performances of the proposed SOP-TENG (nonmoisture) with different electrode sizes. Figure S8: the curves of the normalized voltage of the PET-based TENGs and SOP-TENGs with the same electrode size ((a) 3 × 3 cm^2^ and (b) 3 × 1 cm^2^) at the different relative humidity. Figure S9: study of the effect of the electrode size on the output voltage of TENGs. Figure S10: a schematic diagram of the influence of the electrode size of the PET-based TENG on the absorption of induced charges by water molecules in the air.
