# Supplementary material for: Electron-Ion Coupling Mechanism to Construct Stable Output Performance Nanogenerator
Source: Research (Wash D C). 2021 Nov 9;2021:9817062. doi: 10.34133/2021/9817062 (PMC8600372; doi:10.34133/2021/9817062)
Supplement: Supplementary 1 — Figure S1: fabrication process flowchart of the stable output performance triboelectric nanogenerator (SOP-TENG) based on the composite of the CaCl2-CNF film and the screen-printed electrode. Figure S2: a photograph showing the flexibility of the calcium chloride-cellulose nanofibril (CaCl2-CNF) film. Figure S3: schematic view of the work mechanism of the general hydrogel ionotronic TENG. Figure S4: the voltage output curves of SOP-TENGs at (a) 30 RH%, (b) 50 RH%, (c) 70 RH%, and (d) 90 RH%. Figure S5: the initial normalized voltage, normalized voltage after the swelling, and normalized voltage during the swelling of the SOP-TENG. Figure S6: a comprehensive investigation system for studying the influence of humidity on the electrical output of the SOP-TENG was established, which included a vibration platform, a humidity detection platform, and an electrical measurement platform. Figure S7: electrical output performances of the proposed SOP-TENG (nonmoisture) with different electrode sizes. Figure S8: the curves of the normalized voltage of the PET-based TENGs and SOP-TENGs with the same electrode size ((a) 3 × 3 cm2 and (b) 3 × 1 cm2) at the different relative humidity. Figure S9: study of the effect of the electrode size on the output voltage of TENGs. Figure S10: a schematic diagram of the influence of the electrode size of the PET-based TENG on the absorption of induced charges by water molecules in the air. [file 9817062.f1.docx]

**Supporting Information File**

Electron-Ion Coupling Mechanism to Construct Stable Output Performance Triboelectric Nanogenerator

Yan-Yuan Ba, Jing-Fu Bao, Xin-Tian Liu, Xiao-Wen Li, Hai-Tao Deng, Dan-liang Wen, Xiao-Sheng Zhang*

School of Electronic Science and Engineering, University of Electronic Science and Technology of China, Chengdu 611731, China

*Corresponding Author: [zhangxs@uestc.edu.cn](mailto:zhangxs@uestc.edu.cn) (XS Zhang)

**
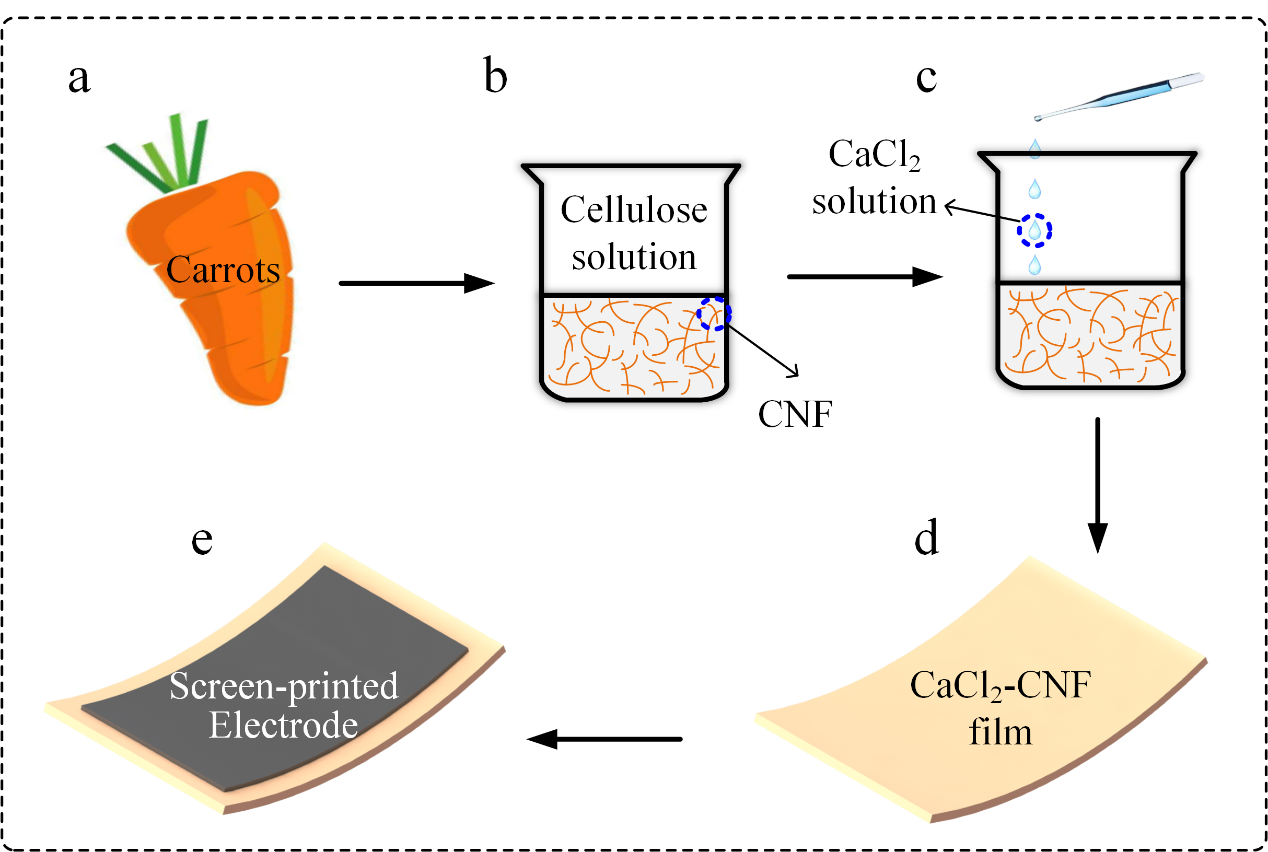
**

**Figure S1.** Fabrication process flow-chart of the stable output performance triboelectric nanogenerator (SOP-TENG) based on the composite of the CaCl_2_-CNF film and the screen-printed electrode.


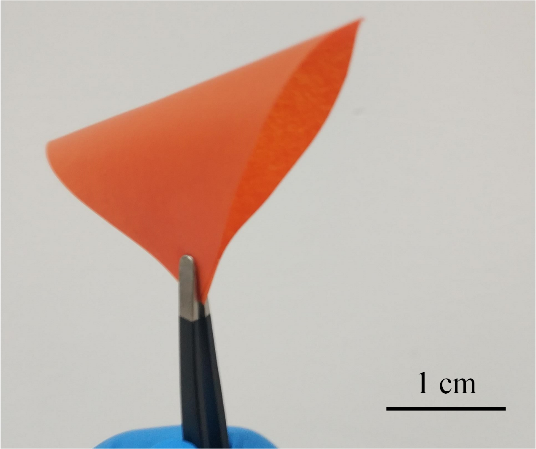


**Figure S2.** A photograph showing the flexibility of the calcium chloride-cellulose nanofibrils (CaCl_2_-CNF) film.

**
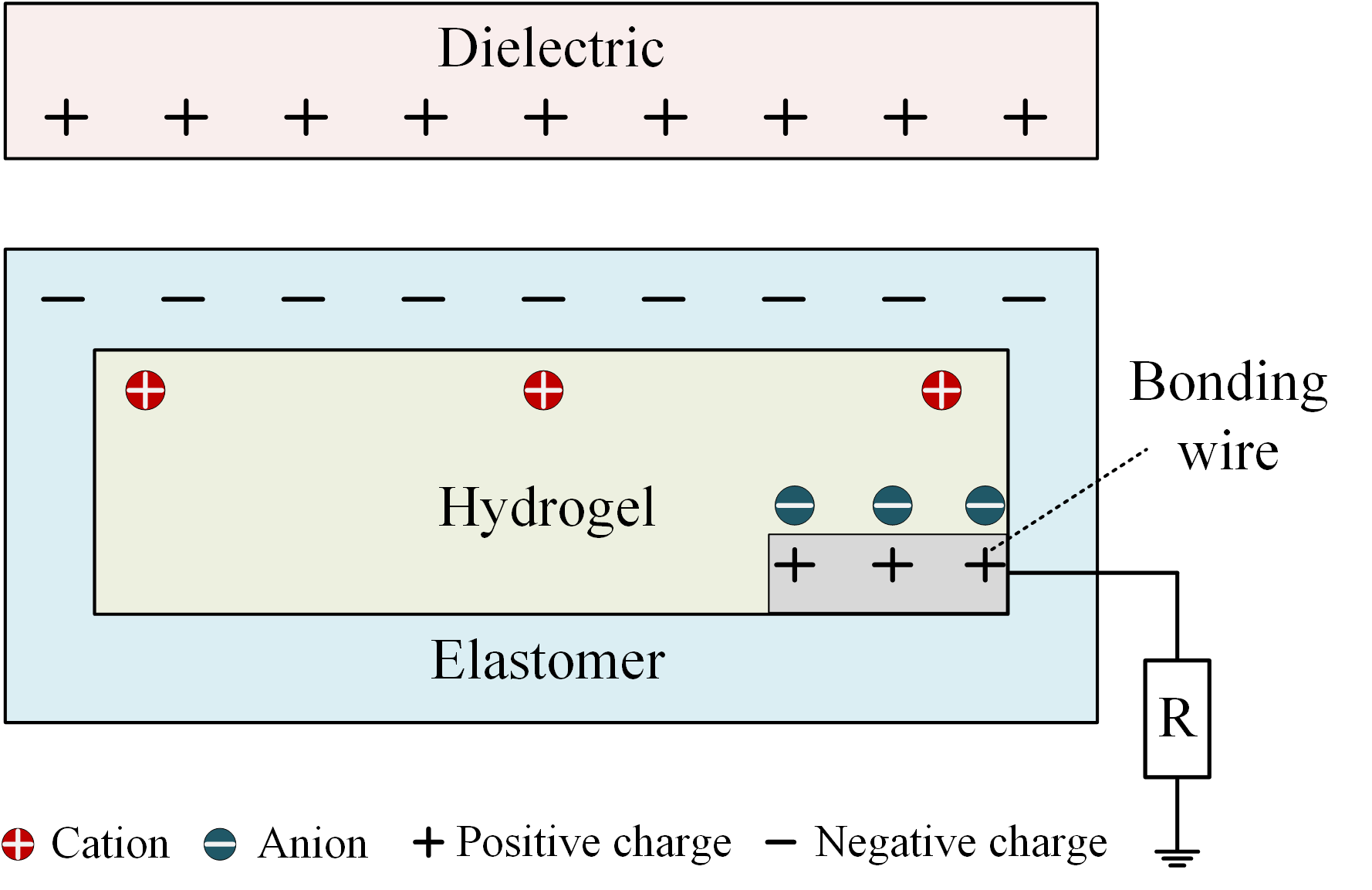
**

**Figure S3.** Schematic view of the work mechanism of the general hydrogel ionotronic TENG. The hydrogel layer which is packaged in an elastomeric cell acts as the electrode, and the elastomeric cell acts as the friction layer.

**
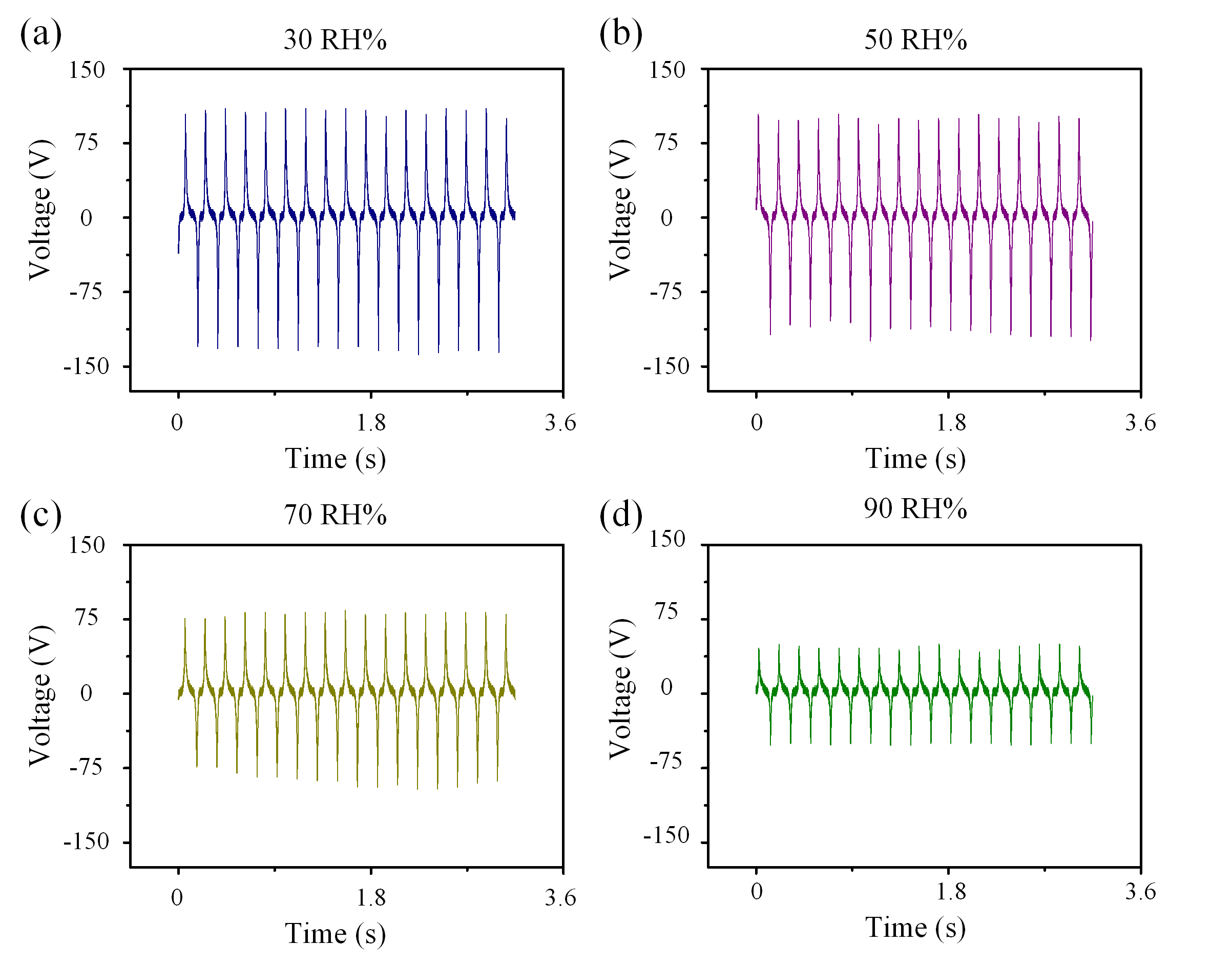
**

**Figure S4.** The voltage output curves of SOP-TENGs at (a) 30 RH%, (b) 50 RH%, (c) 70 RH% and (d) 90 RH%.

**
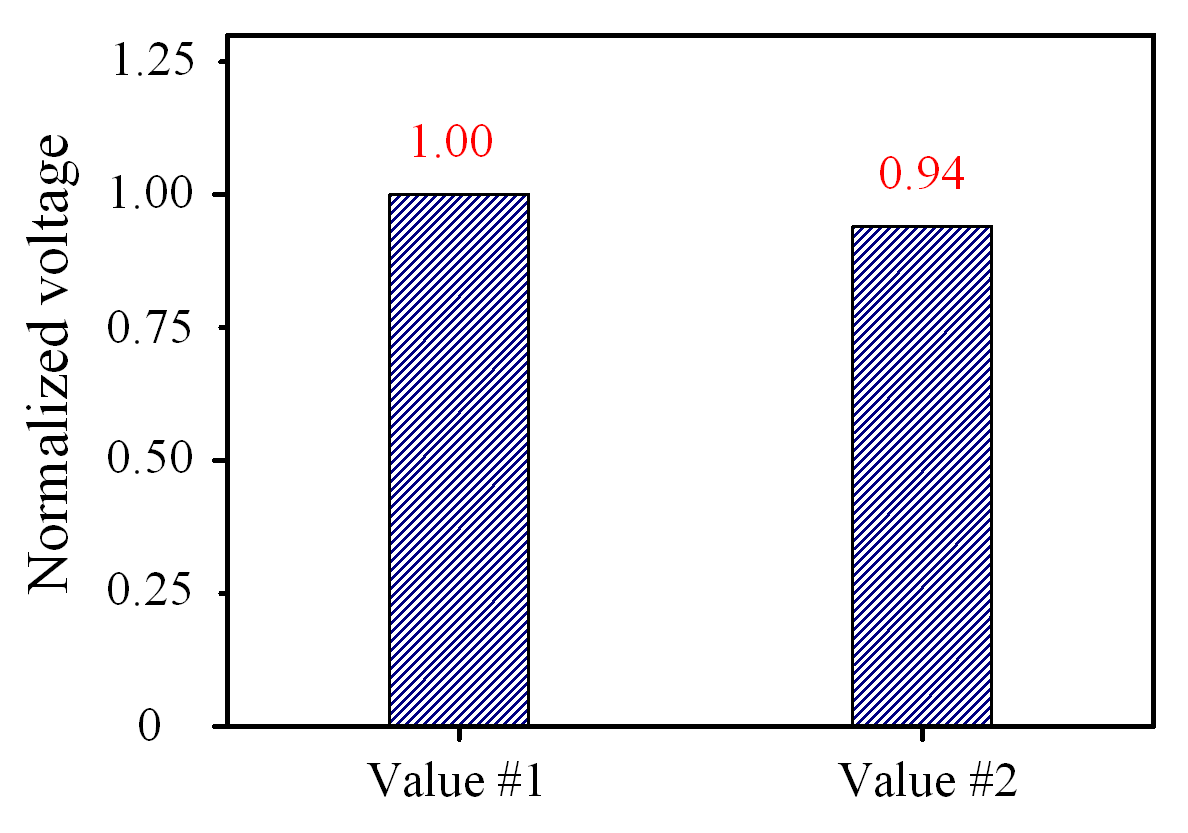
**

**Figure S5.** The initial normalized voltage and normalized voltage after 2 hours of 80 °C drying of the SOP-TENG. Here, Value #1 refers to initial normalized voltage, Value #2 refers to normalized voltage after 2 hours of 80 °C drying of the SOP-TENG.

**
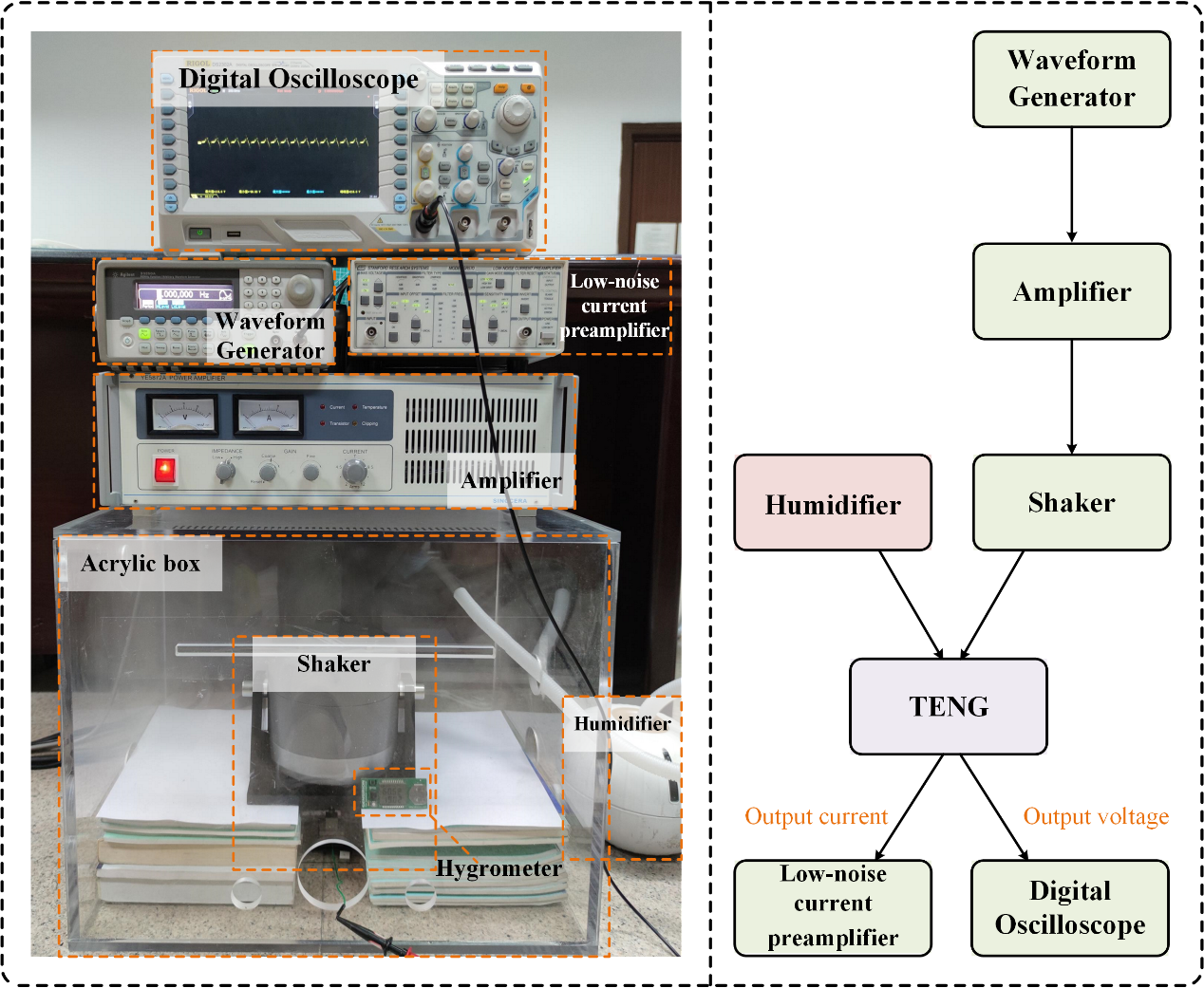
**

**Figure S6.** A comprehensive investigation system for studying the influence of humidity on the electrical output of the SOP-TENG was established, which included a vibration platform, a humidity detection platform and an electrical measurement platform. The vibration platform was built to supply a controllable force with mutative frequency, which included a waveform generator, an amplifier and a shaker. The humidity detection platform was used to measure the relative humidity, which consisted of a humidifier, a hygrometer and an acrylic box. The digital oscilloscope and the low-noise current preamplifier formed the electrical measurement platform to test the voltage and current of the SOP-TENG respectively.


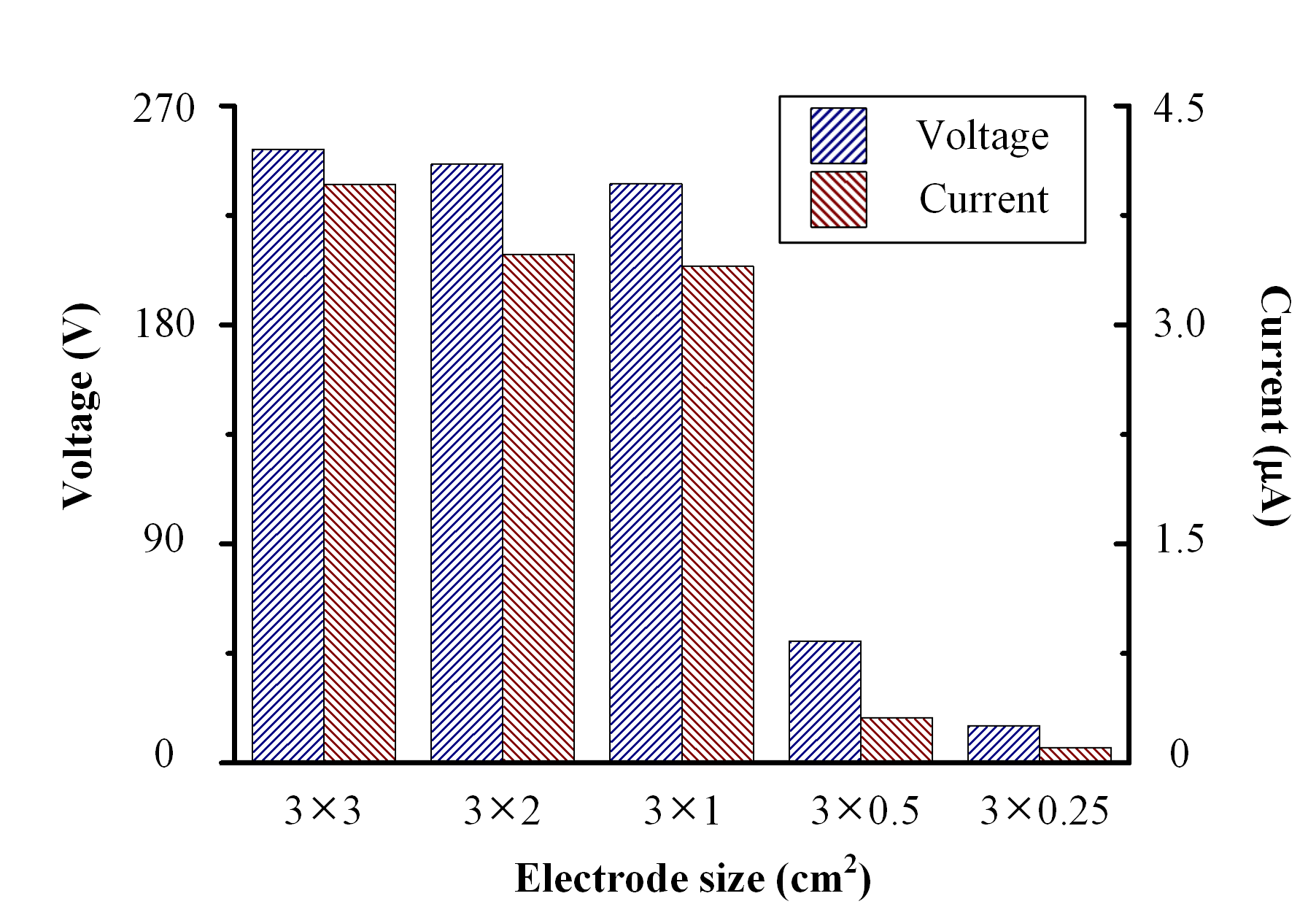


**Figure S7**. Electrical output performances of the proposed SOP-TENG (non-moisture) with different electrode sizes (3×3 cm^2^, 3×2 cm^2^, 3×1 cm^2^, 3×0.5 cm^2^ and 3×0.25 cm^2^). In contrast to SOP-TENG with the 100% size electrode (i.e., electrode area 3×3 cm^2^), it was revealed that SOP-TENG with the 33% size electrode (i.e., electrode area 3×1 cm^2^) possessed almost the same voltage output.

**
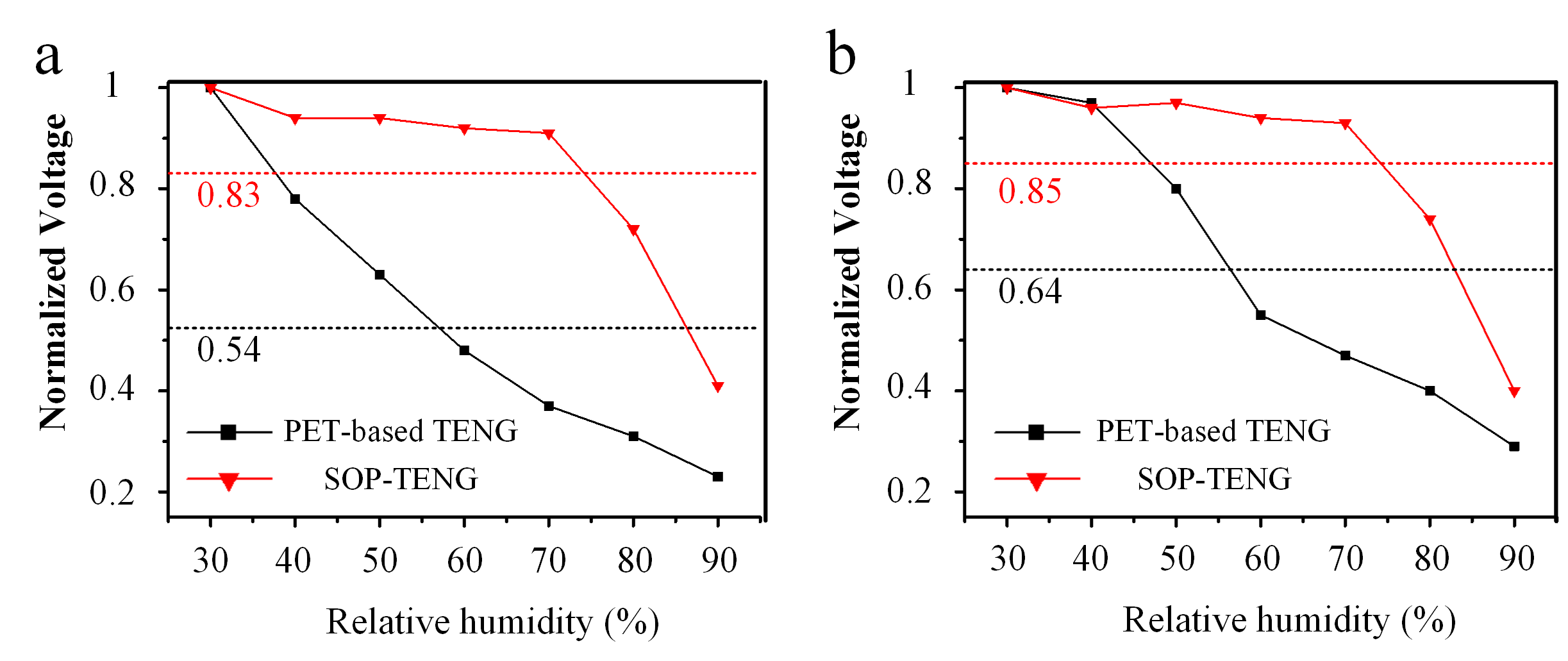
**

**Figure S8.** The curves of the normalized voltage of the PET-based TENGs and SOP-TENGs with the same electrode size ((a) 3×3 cm^2^ and (b) 3×1 cm^2^) at the different relative humidity. Compared to the PET-based TENG, SOP-TENG is not sensitive to the relative humidity of the environment. The reduction of the electrode size will increase the normalized voltage of TENG. The size-reducing by two-thirds increased the average normalized voltage of the PET-based TENG and SOP-TENG by 10% and 2% respectively.

**
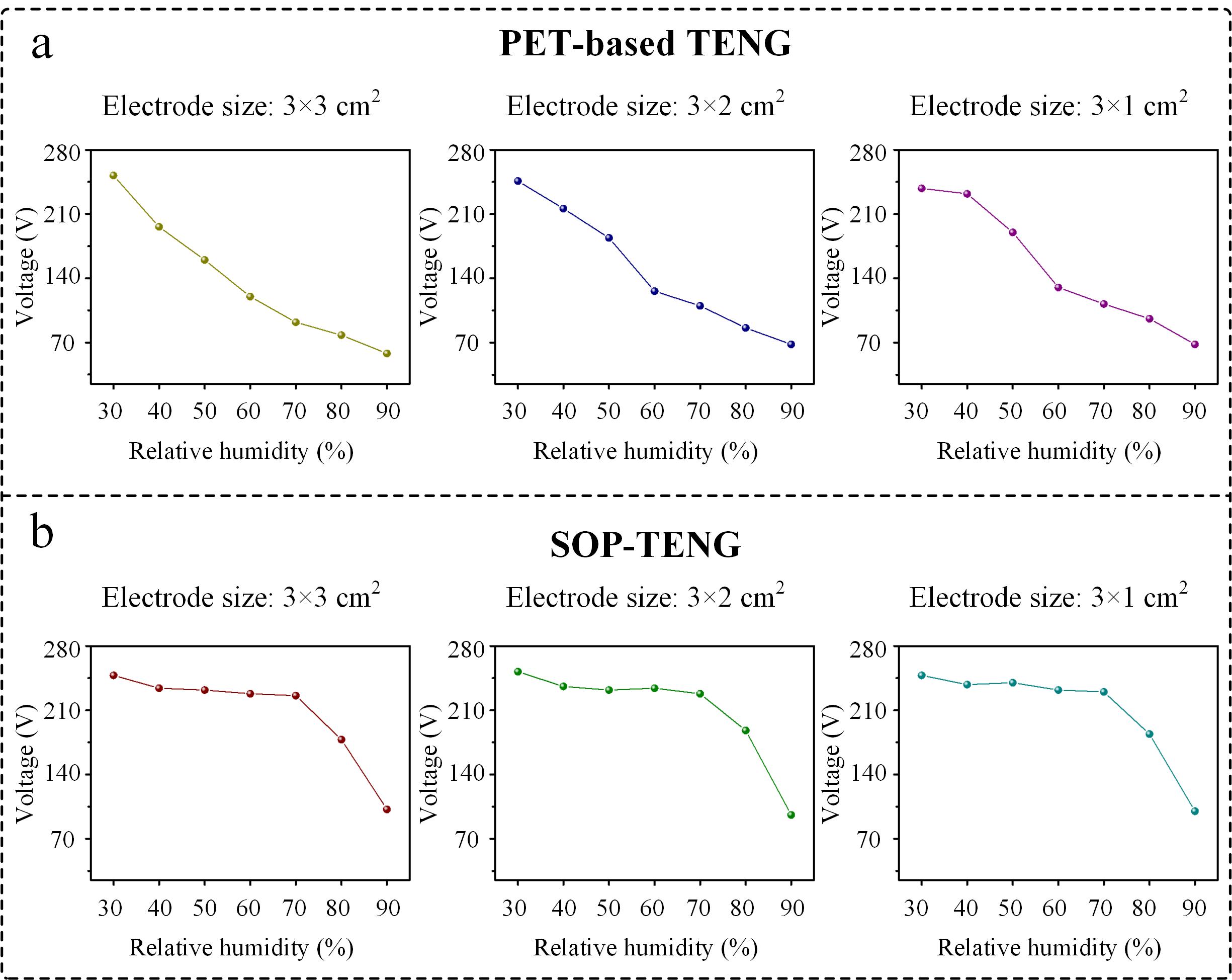
**

**Figure S9.** Study of the effect of the electrode size on the output voltage of TENGs. (a) Effect of the electrode size on the output voltage of the PET-based TENG. The reduction of the electrode size made the voltage curve relatively smoother, although the influence of humidity on the device voltage was still very great. (b) Effect of the electrode size on the output voltage of the SOP-TENG. The reduction of the electrode size had almost no effect on the change of the voltage curve.


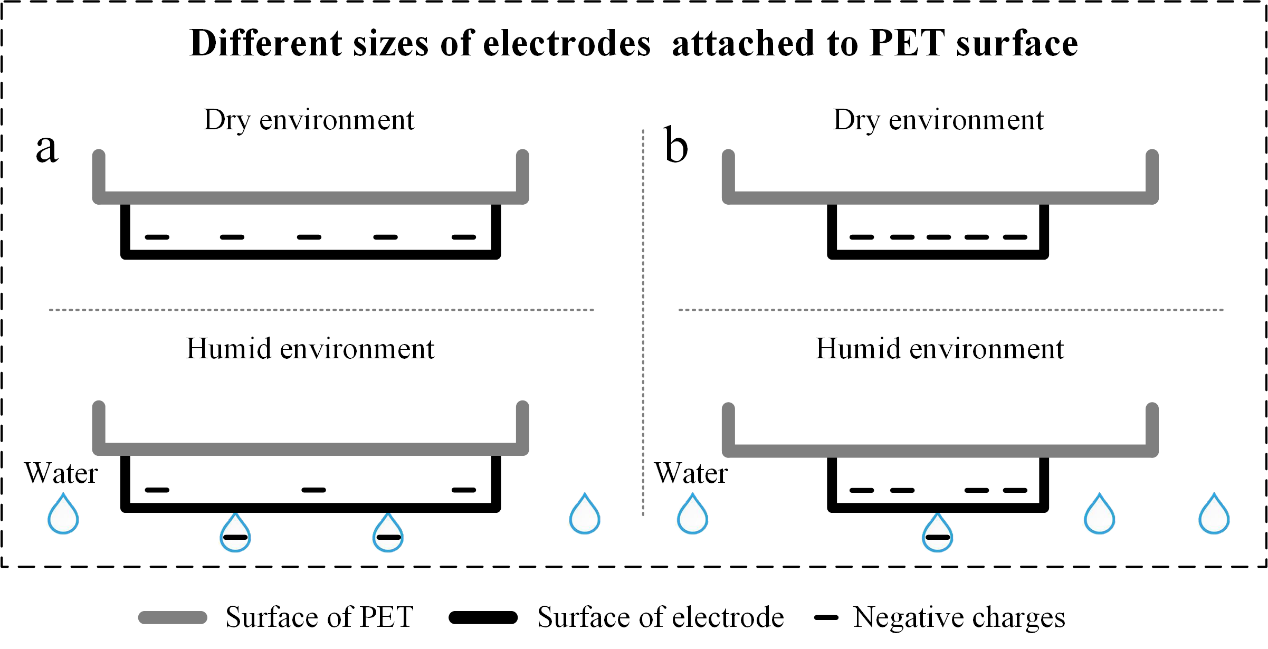


**Figure S10.** A schematic diagram of the influence of the electrode size of the PET-based TENG on the absorption of induced charges by water molecules in the air.

**Supplementary Video S1.** The comprehensive investigation system with a humidity detection platform, a vibration platform and an electrical measurement platform for measuring the output performance of the SOP-TENG at the variational environmental relative humidity.
